# Time for high-burden countries to lead the tuberculosis research agenda

**DOI:** 10.1371/journal.pmed.1002544

**Published:** 2018-03-23

**Authors:** Madhukar Pai

**Affiliations:** 1 McGill International TB Centre & McGill Global Health Programs, McGill University, Montreal, Canada; 2 Manipal McGill Centre for Infectious Diseases, Manipal Academy of Higher Education, Manipal, India

## Abstract

In a Guest Editorial, Madhukar Pai discusses the need for high-burden burden, middle-income countries to take a leading role in tuberculosis research.

At long last, tuberculosis (TB) is getting the political attention that it deserves, being the leading infectious killer of humans today. In 2016, there were 10.4 million estimated new TB cases, with over 1.7 million deaths [1]. The G20 declaration of July 2017 included TB in the context of the need to respond to the antimicrobial resistance threat, following the group’s meeting in Hamburg, Germany [2]. In November 2017, for the first time, a WHO Global Ministerial Conference on TB was held in Moscow, Russia, culminating in the Moscow Declaration to End TB [3]. This year, in September, the United Nations General Assembly (UNGA) will hold the first-ever high-level meeting on the fight against TB [4].

While the political attention brings much needed hope, other developments provide cause for worry. The United States government, the largest funder of TB control and research, is rapidly scaling back on overseas aid, slashing billions from global health and humanitarian assistance [5]. Canada, despite its progressive policies, is spending substantially less on international aid than comparable G7 countries [6]. And while there are considerable uncertainties, Brexit could have a major impact on European Union international development and humanitarian policies and is expected to challenge the EU’s role as the world’s leading donor [7].

The case for funding global health in general, and TB in particular, in this political climate will lack for attention as long as wealthy donor countries focus their priorities on populist and nationalist demands or short-term outcomes of a transactional nature. It is therefore critical for countries most affected by TB to step up, show leadership, and invest in TB control as well as research.

Take the case of Brazil, Russia, India, China, and South Africa (BRICS), which together account for 46% of all incident cases of TB and 40% of all TB-related mortality [1]. With strong, if uneven, economic growth in BRICS, and their growing stature and leadership in the political arena, these countries are well placed to lead the charge against a disease that is a leading killer of their citizens and a huge drain on their economies [8]. In fact, investments in TB control can lead to a huge return on investments for these countries [9].

Commendably, there are signs of the BRICS stepping up to deal with TB, commensurate with their disease burden and economic and scientific prowess [8]. The BRICS Leaders Xiamen Declaration (2017) specifically mentioned the need to improve surveillance of TB and also agreed to set up a TB research network [10].

In fact, the BRICS are now major producers of TB research [11]. While the US remains the top producer of TB research in the past 2 decades, India and China have emerged as the second and third leading producers of TB research in recent years [11]. Further, bibliometric analyses show that the average year-on-year increase in TB publications from the BRICS countries was, in the past decade, nearly double the overall year-on-year increase across all countries [11].

Russia showed leadership in hosting the Global Ministerial Conference, while South Africa has shown commendable initiative in scaling up access to antiretrovirals, as well as new TB diagnostics and drugs (e.g., bedaquiline), and by launching an ambitious National Strategic Plan and 90-90-90 targets to control the linked epidemics of TB and HIV.

Brazil, China, and India have made strong moves to lead on the TB research agenda. In 2015, Brazil established its National TB Research Strategy. In 2016, India launched an India TB Research Consortium to develop new tools for TB and advance evidence-based TB control policies and has increased domestic investments in TB research funding, to align with its National Strategic Plan to Eliminate TB (2017–2025). China is today among the top 5 largest funders of TB research [11]. By making huge domestic investments in science and technology research, China recently overtook the US in terms of total number of science publications [12].

With regards to new tools, both India and China have successfully developed rapid molecular TB tests, using indigenous, more affordable technology platforms, and South Africa is leading the way in vaccine research and clinical trials of new biomarkers as well as TB drug regimens.

While there is evidence of good collaborations between the BRICS and high-income countries (e.g., Indo-US or South Africa-UK collaborations), data suggest that collaborations among BRICS countries are not common [11]. This opportunity must be grasped. As proposed by the BRICS leaders, and reiterated in the Moscow declaration, a TB research network funded and managed by BRICS would go a long way in enhancing collaborations among these countries, improving exchange of technologies and best practices, facilitating multicentric trials of new tools, and avoiding duplication of research efforts.

Along the lines of the BRICS, there is a need for other coalitions of high-burden countries to step up. About 25% of the global incident cases occur in the WHO African Region, with the proportion of TB cases coinfected with HIV exceeding 50% in parts of southern Africa [1]. In this context, the African Union, which consists of all 55 countries on the African continent, is well placed to take leadership on the TB-HIV coepidemic response.

Eliminating TB will not be possible without new tools and approaches. In the Moscow Declaration to End TB, more than 120 national delegations committed to developing and implementing more ambitious, fully funded national TB policies and strategic plans, including for TB research, that are aligned with national health plans and consistent with the End TB Strategy [3]. It is critical that all stakeholders work together to make this declaration a reality and hold political leaders accountable for their pledges, especially their commitment to increased domestic funding to ensure quality TB care and social protection for the most vulnerable sections of the population.

The world cannot depend on a few wealthy countries with very low TB incidence to support all the research that is required to tackle TB. High-burden, middle-income countries with high TB rates must step up. They have the potential to transform the global TB research agenda through increased domestic funding, collaborative networks, and transnational research partnerships. By taking the lead on TB research, high-burden countries not only can meet their own national strategic plan goals but can also take a leading step towards fulfilling the commitment to end the TB epidemic, with targets to reduce TB deaths by 95% and to reduce TB incidence rate by 90% between 2015 and 2035.

## Abbreviations

BRICS: Brazil, Russia, India, China, and South Africa
TB: tuberculosis
UNGA: United Nations General Assembly
